# Exploiting Base Excision Repair to Improve Therapeutic Approaches for Pancreatic Cancer

**DOI:** 10.3389/fnut.2015.00010

**Published:** 2015-03-27

**Authors:** George Sharbeen, Joshua McCarroll, David Goldstein, Phoebe A. Phillips

**Affiliations:** ^1^Pancreatic Cancer Translational Research Group, Lowy Cancer Research Centre, Prince of Wales Clinical School, UNSW Australia, Sydney, NSW, Australia; ^2^Children’s Cancer Institute, Lowy Cancer Research Centre, UNSW Australia, Sydney, NSW, Australia

**Keywords:** base excision repair, pancreatic cancer, chemoresistance, therapeutic targets, prognostic factors

## Abstract

Pancreatic ductal adenocarcinoma (PDA) is a highly chemoresistant and metastatic disease with a dismal 5-year survival rate of 6%. More effective therapeutic targets and approaches are urgently needed to tackle this devastating disease. The base excision repair (BER) pathway has been identified as a predictor of therapeutic response, prognostic factor, and therapeutic target in a variety of cancers. This review will discuss our current understanding of BER in PDA and its potential to improve PDA treatment.

## Introduction

Pancreatic ductal adenocarcinoma (PDA) currently ranks as the fourth leading cause of cancer-related death in Western societies (1–3) and is projected to become the second leading cause by 2030 (4). Surgery is currently the best chance for a cure, but <15% of patients present with operable disease due to the lack of specific symptoms (2, 3). The remaining patients that present with unresectable tumors have a dismal prognosis, with an overall 5-year survival rate at 6% (5). This poor prognosis is largely due to the highly metastatic and chemoresistant nature of PDA (6, 7). Recent years have seen some improvement in treatments for PDA. Gemcitabine/abraxane combination therapy is a current standard of care for unresectable PDA, but this only extends median overall survival by 8 weeks over gemcitabine monotherapy (8). More aggressive polychemotherapeutic regimens, such as FOLFIRINOX, are also employed as treatments for PDA but only extend median survival by 17 weeks over gemcitabine monotherapy (9) and require careful selection of candidates for the treatment due to toxicity. There is clearly an urgent need for novel therapeutic approaches if we are to achieve a significant improvement in PDA patient survival.

## A Mutagenic Environment: Impact on DNA Repair

Pancreatic ductal adenocarcinoma is an epithelial-derived tumor that is proposed to proceed in a step-wise manner from pancreatic intraepithelial neoplasia (PanIN) lesions to PDA (10). PDA tumors are highly fibrotic and consist of a complex microenvironment containing PDA cells, cancer-associated pancreatic stellate cells (CA-PSCs), immune cells, and extracellular matrix proteins (11–13). CA-PSCs play a major role in generating the extensive fibrotic response that is characteristic of PDA, by secreting excessive amounts of extracellular matrix proteins in response to signals from PDA cells (11, 12, 14–18). The fibrotic PDA microenvironment drives the highly chemoresistant and metastatic phenotype of this cancer. Fibrosis distorts the tumor vasculature, creating a hypoxic and nutrient-deprived microenvironment (11, 19–21). These two features are known to drive the Warburg effect, which is a switch from oxidative to glycolytic metabolism, as well as the transition of cancer cells from an epithelial phenotype to a more aggressive mesenchymal phenotype (22–25).

**Figure 1:**
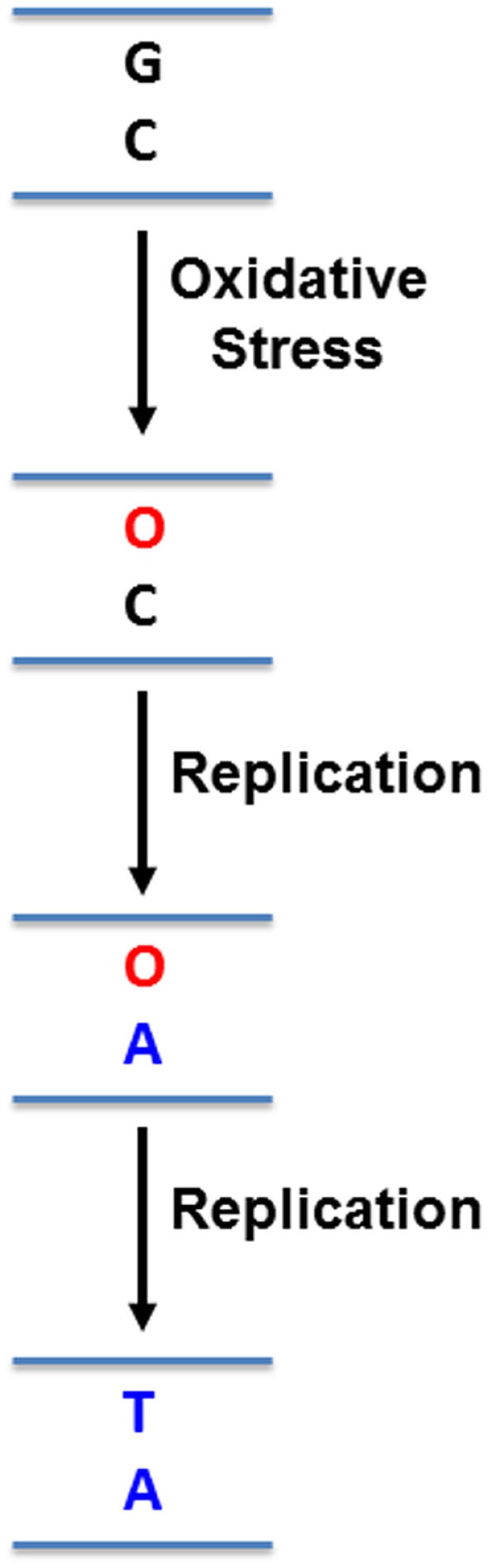
**Mutation induced by 8-oxo-guanine**. Reactive oxygen species can oxidize guanines (G) in DNA to 8-oxo-guanine (O). Replication machinery can then mis-insert adenine (A) opposite O. In a subsequent round of replication, a thymine (T) can be inserted opposite A, resulting in a G:C to T:A mutation.

While this microenvironment selects for more aggressive pancreatic cancer cells, it also imposes mutagenic pressure on the cells, particularly in the form of oxidative stress (24, 26, 27). Hypoxia triggers the release of reactive oxygen species from mitochondria, initiating a signaling cascade that reprograms the cell to facilitate its survival (26, 27). In addition, the Warburg effect results in loss of potent anti-oxidant intermediates, reducing the ability of cells to deal with increased intracellular oxidative stress (24). These free radicals readily react with DNA bases, altering how DNA polymerases might recognize them. For example, the most common form of DNA damage induced by reactive oxygen species is 8-oxo-guanine (8-oxo-G) in guanine:cytosine (G:C) base pairs (28) (Figure 1). If left unrepaired, replication machinery can mis-insert adenine (A) opposite 8-oxo-G (29) (Figure 1). In subsequent rounds of replication, this can permanently convert a G:C base pair to a thymine:adenine (T:A) base pair (29) (Figure 1). Rapidly proliferating PDA cells must be able to tolerate this increased mutagenic load in order to replicate their genome, without accumulating genomic damage that can disrupt its most basic survival functions. Under these conditions, effective DNA repair becomes critical for PDA cell survival. The base excision repair (BER) pathway is responsible for repair of a variety of damaged DNA bases, and plays a prominent role in repair of oxidative DNA damage, which is particularly relevant to PDA as outlined above (30). Importantly, components of this pathway have increasingly been identified as predictors of cancer risk, prognosis, chemoresistance, and as direct therapeutic targets in a variety of cancers (31). This review will discuss components of the BER pathway that have been identified as therapeutic targets and predictors of therapeutic response in pancreatic cancer. It will also discuss the untapped potential for other PDA therapeutic targets in this pathway.

## The Base Excision Repair Pathway

**Figure 2:**
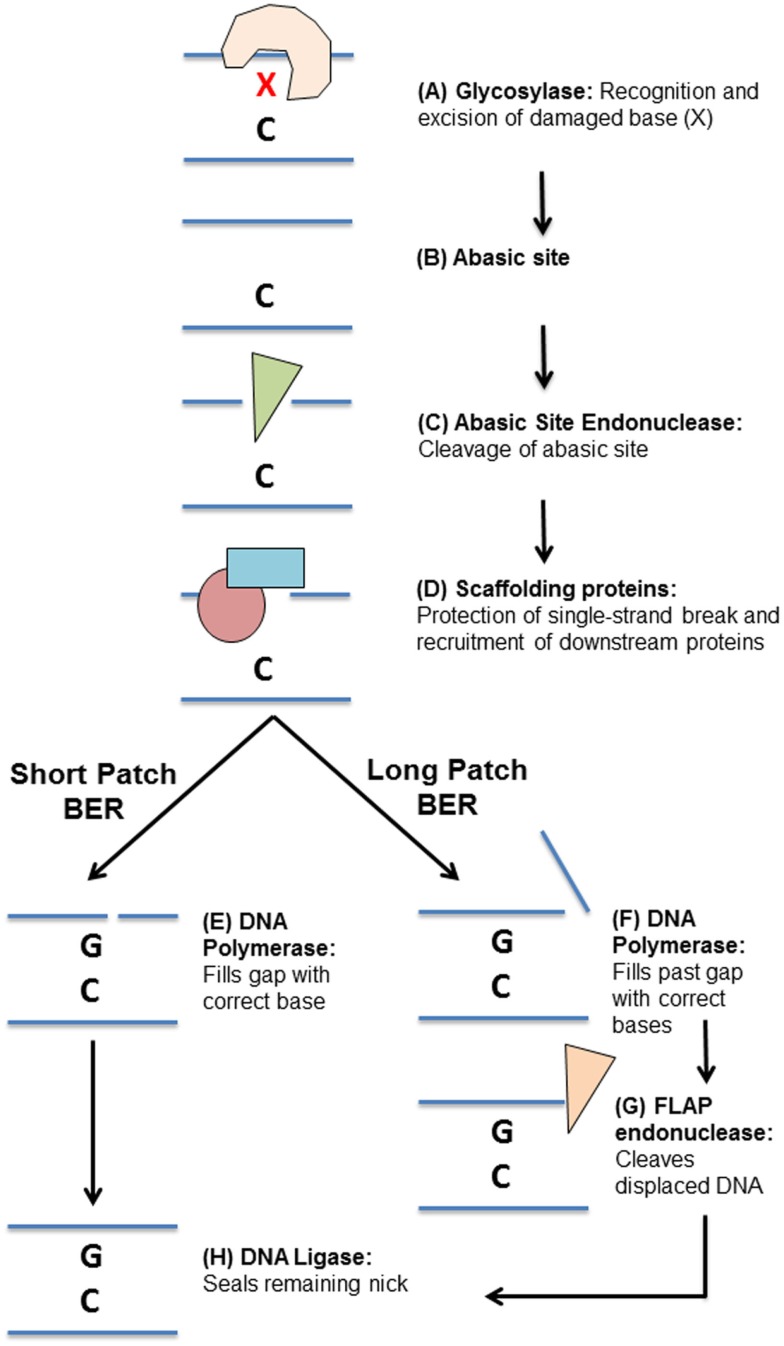
**Overview of the base excision repair pathway**. **(A)** DNA damage “X” is detected and excised by a specific glycosylase leaving an abasic site **(B)**. **(C)** The abasic site processed by an apurinic/apyrimidinic endonuclease. **(D)** Scaffolding proteins bind the single-stranded DNA and recruit downstream base excision repair proteins. Repair can then proceed by **(E)** short-patch or **(F)** long-patch base excision repair. **(E)** A DNA polymerase fills in the missing DNA base. **(F)** DNA polymerases fill in the missing DNA base and continue synthesizing DNA past the initial damage site, displacing the original DNA strand. **(G)** A flap endonuclease then cleaves the flap of single-stranded DNA. **(H)** A DNA ligase seals the remaining DNA nick, completing the repair.

The BER pathway consists of a number of specialized glycosylases, endonucleases, polymerases, and ligases that work together to repair a variety of damaged DNA bases (Figure 2). Generally speaking, BER of DNA damage proceeds through five steps. The first step is detection and removal of the damaged DNA base, which is carried out by glycosylases (Figure 2A). Glycosylases can be largely grouped by the types of damaged bases that they recognize and remove (Table 1): uracil/thymine excision enzymes (UNG, SMUG1, TDG, and MBD4) (32–37), 8-oxo-G repair enzymes (OGG1 and MYH) (38–45), oxidized pyrimidine repair enzymes (NTH1 and NEIL1–3) (46–56), and methyl-purine glycosylase (MPG) (57–59) (Table 1). Their substrates include many of the damaged bases generated by DNA-damaging chemotherapeutics (31).

**Table 1:** **Base excision repair proteins and function**.

Group	Protein name	Synonym	Function in base excision repair
Uracil/thymine glycosylases	Methyl-CpG-binding domain protein 4	MBD4	Binds methylated DNA and excision of 5-hydroxymethyluracil
	Single-strand selective monofunctional uracil DNA glycosylase	SMUG1	Excision of uracil
	Thymine DNA glycosylase	TDG	Excision of mismatched thymines and uracils
	Uracil DNA glycosylase	UNG	Excision of uracil
8-Oxo-G repair glycosylases	MutY homolog	MYH	Excision of A mispaired with 8-oxo-G
	8-Oxo-G glycosylase 1	OGG1	Excision of 8-oxo-G
Oxidized pyrimidine glycosylases	n^th^ endonuclease III-like 1	NTH1	Excision of oxidized pyrimidines
	Endonuclease VIII-like 1–3	NEIL1–3	Excision of oxidized pyrimidines
Methyl-purine glycosylase	Methyl-purine-DNA glycosylase	MPG	Excision of methyladenine and methylguanine
Abasic site cleavage and processing	Apurinic/apyrimidinic endonuclease 1	APE1	Cleavage of abasic sites
	Polynucleotide kinase 3′-phosphatase	PNKP	Processing of cleaved abasic sites left by NEIL glycosylases
Scaffolding proteins	Poly ADP ribose polymerase-1	PARP1	Protection of DNA breaks and recruitment of XRCC1
	X-ray repair cross-complementing protein 1	XRCC1	Recruitment of base excision repair proteins downstream of abasic site cleavage
DNA polymerases	DNA polymerase beta	POLB	Re-synthesis of DNA
DNA ligases	DNA ligase I	LIGI	Sealing of DNA nick
	DNA ligase III	LIGIII	Sealing of DNA nick

Once a glycosylase has removed a damaged base, it leaves behind a non-instructional abasic site (Figure 2B). This site can cause transcription stalling and must be cleaved and processed before repair can continue (60, 61). The second step in BER is thus cleavage of the abasic site (Figure 2C). Some glycosylases are bifunctional, meaning they can cleave the abasic site that they create (62–65). However, most glycosylases require cleavage of the abasic site by apurinic/apyrimidinic endonuclease 1 (APE1) (66, 67). Processing of an abasic site leaves a single-strand break, which needs to be protected to prevent further damage.

The third step in BER is binding of the single-strand break by scaffolding proteins (Figure 2D). There are two major scaffolding proteins in BER, poly ADP ribose polymerase-1 (PARP1) and X-ray repair cross-complementing protein 1 (XRCC1) (68–72). Once these proteins are bound to the single-strand DNA break, they recruit other BER proteins required to complete the repair (68–72).

The fourth step in BER is insertion of the missing base by a DNA polymerase, such as DNA polymerase β (POLB) (73). This can take the form of either single base repair, whereby only one base is inserted (short-patch repair, Figure 2E), or long-patch repair (Figure 2F), where a stretch of bases is inserted at and beyond the initial site of damage, displacing some of the nearby bases (74). In long-patch BER, an additional step is required involving the specialized flap-endonuclease, FEN1, to cleave the displaced stretch of DNA bases (Figure 2G) (75). The final step in BER is then sealing of DNA nicks left by these processes DNA ligase I (LIGI, long-patch BER) or DNA ligase III (LIGIII, short-patch BER) (Figure 2H) (76).

Cancer cells take advantage of BER’s ability to repair DNA damage in order to resist DNA-damaging chemotherapies and radiotherapy (31). BER proteins have thus been identified as potential therapeutic targets and chemoresistance factors in a variety of cancers (31). More specifically in PDA, the BER proteins APE1, XRCC1, and PARP1 provide well-studied examples of how BER proteins can be applied as therapeutic targets or predictors of therapeutic response (detailed below). They also highlight the complex roles that BER proteins can play outside of their primary DNA repair roles in the BER pathway, which can be exploited in therapeutic approaches to elicit a broader impact in PDA cells.

## APE1 as a Therapeutic Target for Pancreatic Cancer

APE1 is an endonuclease that cleaves DNA abasic sites left by the activity of glycosylases (66, 67). This makes APE1 a central protein in the BER pathway, as its activity is required downstream of a variety of glycosylases. It is therefore not surprising that APE1 has been implicated in resistance to a diverse range of therapeutics. Moreover, APE1 can directly repair the replication-blocking abasic sites generated by alkylating agents (77). An analysis of APE1 activity in medulloblastomas and neuroectodermal tumors from patients treated with adjuvant radiation and multi-agent chemotherapy, found that increased APE1 activity correlated with poorer response to treatment (78). The same association has been observed in grade II–III gliomas, whereby increased APE1 activity correlated with increased resistance to radiation therapy and alkylating agents (79). Similar trends have been observed in head-and-neck cancer in relation to resistance to chemotherapy and radiotherapy (80). APE1 can also confer resistance to chemotherapeutics that may not directly generate substrates for the BER pathway, but may generate secondary types of DNA damage that BER is required to resolve. For example, APE1 upregulation has been associated with platinum resistance in ovarian cancer (81). This is likely due to the generation of reactive oxygen species by platinum-based therapies, which generates oxidized DNA bases that require BER to repair (82).

APE1’s involvement in chemoresistance and radioresistance in cancer cells has been functionally demonstrated using small molecule inhibitors and siRNA-based approaches. APE1 inhibitors have been shown to enhance the sensitivity of HeLa cells to the alkylating agent methyl methanesulfonate (MMS) (83). In an siRNA-based approach, APE1 silencing has been shown to increase the chemosensitivity of ovarian cancer cells to cisplatin via induction of apoptosis (81). APE1 inhibition has similarly been shown to sensitize PDA cells (Panc-1) to gemcitabine (84), while Xiong et al. (85) later reproduced these findings in SW1990 PDA cells. APE1 downregulation has since been shown to also radiosensitize PDA cells (86).

APE1 inhibition also highlights how BER proteins can represent broader intracellular targets that can both chemosensitize cancer cells and compromise cancer cell survival. APE1 is also known as Ref-1, as its N-terminal plays a role as a redox factor, responsible for regulating a variety of transcription factors that facilitate cell survival in response to oxidative stress (87–92). Several studies have identified APE1 as a survival factor in PDA cells. Jiang and colleagues (93) observed that APE1 silencing in PDA cells (Panc1 and MiaPaCa2), reduced proliferation/colony forming ability and increased apoptosis, by increasing DNA damage. Studies have similarly demonstrated this using a small molecule inhibitor of APE1 (E3330) (94, 95). Fishel et al. (94) showed that E3330 reduced PDA cell and cancer-associated endothelial cell proliferation and migration, decreased transcription factor activity for NFκB, AP-1, and HIF-1α, and reduced tumor growth in PDA xenograft mouse models. Recently, Fishel et al. (96) further demonstrated the complexity of signaling networks involving APE1. The group identified a novel interaction specifically between the redox component of APE1 and nuclear factor erythroid-related 2 (Nrf2) in PDA cells. Inhibition of APE1 using small molecule and genetics approaches increased activation of Nrf2, a protein known to play a role in protection from oxidative stress (96). This work identified a potential resistance mechanism that would need to be co-targeted with APE1 inhibitors. Cardoso and colleagues (95) demonstrated that dual targeting of APE1 and the transcription factor STAT3 synergistically reduced PDA cell survival and migration. The authors demonstrated that STAT3 DNA binding and transcriptional activity is under the control of APE1 (95), suggesting that the synergistic effects may have been due to enhancement of STAT3 inhibition by knocking-down APE1, or by inhibiting functions of each protein that were independent of the other, in addition of their co-dependent pathway. This also provides an important example of how the complex signaling roles of some BER proteins can have far reaching effects on how PDA cells interact with their microenvironment and migrate.

**Figure 3:**
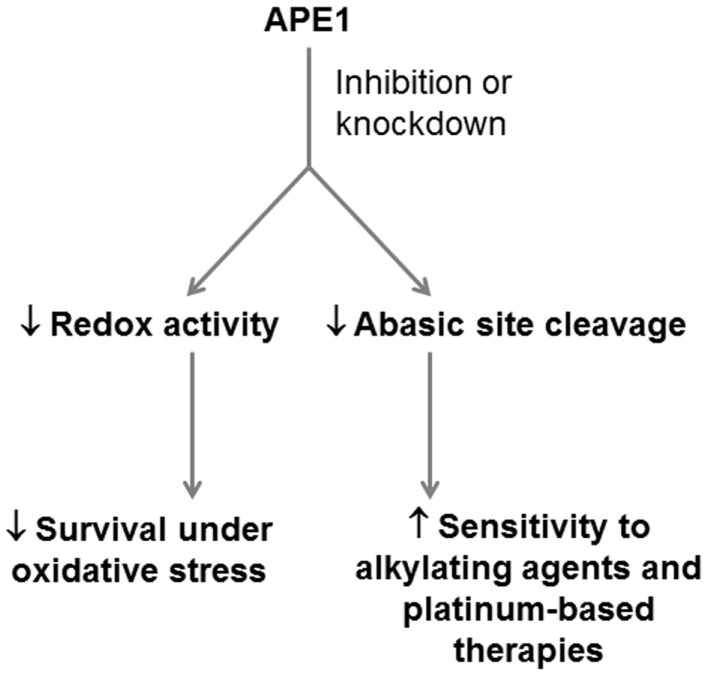
**Potential application of APE1 as a therapeutic target in PDA**. APE1 represents a potential dual target in PDA, whose inhibition can reduce the ability of PDA cells to respond to oxidative stress through its redox signaling role, and the ability of PDA cells to resist alkylating agents and platinum-based drugs through its role as an abasic site endonuclease.

As a therapeutic target for PDA, APE1 is ideal in its ability to confer sensitivity to a variety of drugs and to broadly interfere with the cellular response to oxidative stress (Figure 3). However, this involvement in a broad range of BER sub-pathways also makes systemic inhibition of APE1 risky, as off-target toxicity and the risk of generating cancers elsewhere in the body would not be unexpected. APE1 knockout is embryonic lethal (97, 98) and it has been demonstrated to be essential for mammalian cell survival (99, 100). An approach targeting APE1 should preferably be cancer cell-specific, for example, siRNA complexed to a nanoparticle, which has cancer cell-specific targeting moieties attached (101, 102).

## XRCC1 and Chemotherapeutic Response in Pancreatic Cancer

XRCC1 is a scaffolding protein with no enzymatic activity, which plays an important role in recruiting and coordinating other BER proteins at sites of DNA damage (68–72). XRCC1 polymorphisms that interfere with PARP binding and cause broadly reduced BER efficiency have been implicated in resistance to platinum-based therapies in lung and colorectal cancer (103–105). Interestingly, these are an example of how *reduced* DNA repair efficiency can confer resistance to chemotherapeutics. This relationship might appear illogical as DNA adducts generated by platinum therapies are not direct targets of the BER pathway and are instead processed by the nucleotide excision repair pathway (106). However, studies by Kothandapi and colleagues (107, 108) provide a potential explanation for this relationship and highlight the complex interaction of DNA repair pathways. The study suggested that rather than acting directly on the primary DNA damage, that is, DNA inter-strand cross-links, or adducts, BER could act on DNA that was exposed and deaminated around platinum-induced DNA adducts (107, 108). However, BER at these sites would interfere with the nucleotide excision repair pathway that directly repairs the DNA adducts, thus maintaining platinum-induced toxicity (107, 108). XRCC1 polymorphisms that reduce BER recruitment to these sites would reduce interference with the nucleotide excision repair pathway, allowing more effective repair of the DNA adduct and increasing cancer cell resistance to the drug.

There is also evidence that lower XRCC1 expression is associated with increased sensitivity to platinum-based therapies in ovarian cancer cells, as demonstrated by increased accumulation of DNA double-strand breaks in response to platinum-based therapies (109). While these results may appear to be conflicting with the studies presented so far, they actually highlight two important points that we need to consider to effectively use BER proteins as a predictor of therapeutic response: (i) the biochemistry of the DNA damage induced by a therapeutic and (ii) cross-talk between DNA repair pathways that respond. In this case, we need to note that XRCC1 is also required for the final stages of the nucleotide excision repair pathway, which *directly* repairs the toxic DNA adducts generated by platinum drugs (110, 111). Thus, decreased XRCC1 interferes with the ability of nucleotide excision repair to remove the DNA adducts that facilitate cell death. On the other hand, polymorphisms that hinder recruitment of XRCC1 through the BER pathway reduce this interference with nucleotide excision repair and increase resistance to platinum-based therapies.

XRCC1 polymorphisms can similarly be applied in PDA to predict response to platinum-based drugs and to identify individuals with increased risk of pancreatic cancer incidence. In particular, a polymorphism in Arg399 of XRCC1 has been identified in pancreatic cancer studies as a risk factor and predictor of therapeutic response (112–114). This polymorphism occurs within a PARP binding domain of XRCC1 and has been associated with decreased BER function (112). Giovanetti and colleagues (113) identified a significant correlation between the Arg399 polymorphism of XRCC1 and a worse response to platinum-based therapeutic regimens (113), indicating that XRCC1 could be used in PDA to predict response to platinum-based therapies as in other cancers.

Moreover, XRCC1 polymorphisms can be used to predict PDA risk. Duell and colleagues (112) observed that carriers of this XRCC1 polymorphism who were smokers, had significantly higher risk of developing PDA than other members in the cohort of 309 PDA patients and 964 control individuals. Nakao et al. (114) similarly found a significant correlation between the XRCC1 Arg399 polymorphism and increased pancreatic cancer risk in a cohort that included 185 Japanese pancreatic cancer patients. As mentioned earlier, BER plays a major role in repairing oxidative DNA damage (30). Oxidative DNA damage occurs as a consequence of normal cell metabolism and can be increased by environmental factors, especially smoking (115–118). The increased pancreatic cancer risk in individuals carrying the Arg399 polymorphism of XRCC1 is potentially the result of a reduced cellular capacity to repair mutations that result from oxidative DNA damage. Thus, the XRCC1 Arg399 polymorphism, in combination with environmental factors, could potentially be used to identify high-risk individuals for early pancreatic cancer screening.

**Figure 4:**
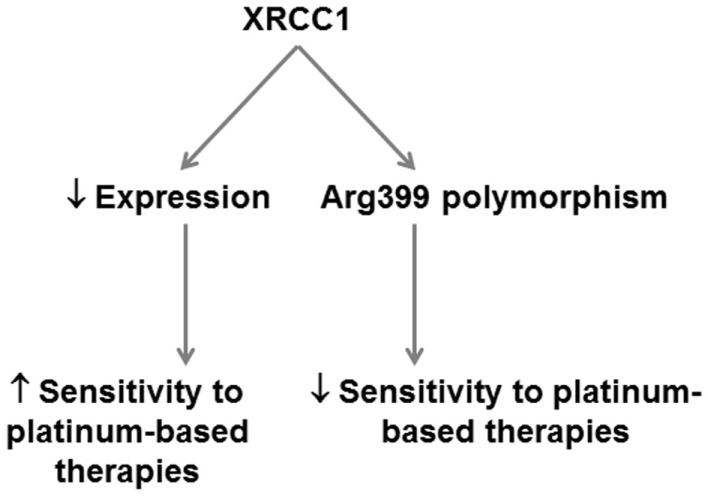
**Potential application of XRCC1 as a predictor of therapeutic response in PDA**. XRCC1 expression and polymorphisms can potentially be applied in PDA to predict therapeutic response. XRCC1 downregulation predicts increased sensitivity to platinum-based therapies. XRCC1 Arg399 polymorphisms that decrease base excision repair efficiency predict poorer response to platinum-based therapies.

While the development of PARP inhibitors (discussed in next section) removes some incentive to therapeutically target XRCC1, this protein clearly represents an important predictive factor for cancer risk and platinum-based therapeutics in PDA (Figure 4).

## PARP1 as a Therapeutic Target for Pancreatic Cancer

Poly(ADP)-ribose polymerase-1 binds single-strand and double-strand DNA breaks and interacts with XRCC1 to help recruit downstream BER proteins to facilitate repair (68, 72). However, unlike XRCC1 it can also directly regulate proteins, particularly histones and transcription factors, by adding poly(ADP)-ribose units to them (termed PARylation), which it synthesizes from NAD+ (119). PARP uses this activity to rapidly co-ordinate DNA repair in response to the DNA breaks (119). PARP inhibitors are the perfect example of how an understanding of BER and its interaction with other pathways can be effectively applied to tailor therapeutic approaches for cancer. This field was launched by two landmark studies published in 2005 (120, 121) demonstrating that PARP inhibition was an effective approach to treat BRCA1- and BRCA2-defective cancer cells (cells defective in homologous recombination). These studies not only identified a new therapeutic target but also highlighted the potency of “synthetic lethality” approaches – approaches that compound existing weaknesses in DNA repair in cancer cells by eliminating complimentary repair pathways, thus overwhelming cells with DNA damage. BRCA1/2-deficient cells are unable to carry out effective homologous recombination to repair DNA double-strand breaks (122). Inhibition of PARP-1 eliminates the remaining active DNA repair pathways that can respond to these breaks and potentially generates more breaks by inhibiting multiple DNA repair pathways. PARP inhibitors are now applied in the clinic for BRCA1/2 defective ovarian and breast cancers and are in clinical trial for a variety of other BRCA1/2-defective tumors (123).

Pre-clinical studies in PDA cells have similarly demonstrated the efficacy of PARP inhibitors in synthetic-lethal approaches and in sensitization of PDA cells to therapeutics. Drew and colleagues (124) demonstrated that BRCA2-defective PDA cells (CAPAN-1 line) were sensitive to the PARP1 inhibitor AG014699 *in vitro* and in sub-cutaneous mouse tumors. More recently, Chen et al. (125) combined inhibition of PARP family proteins by olaparib with inhibition of Bcl-2, an anti-apoptotic protein that is now recognized as a suppressor of non-homologous end-joining, which is an alternate pathway for double-strand DNA break repair (126, 127). This drug combination synergistically caused growth arrest and non-apoptotic cell death in PDA cells *in vitro* and in PDA xenografts (125). A possible explanation for these synergistic effects might be that PARP inhibition increases the persistence of double-strand breaks while Bcl-2 inhibition increases erroneous joining of DNA double-strand breaks by the non-homologous end-joining pathway, eventually leading to impairment of cell function by accumulation of translocations and mutations.

PARP inhibition need not only be applied on BRCA1/2-defective backgrounds but can also improve the efficacy of DNA-damaging agents by the same principle. For example, Jacob et al. (128) showed that PARP inhibition using 3-aminobenzamide (an inhibitor that competes with NAD+), could enhance the efficacy of gemcitabine in PDA cells. In an approach that indirectly targeted PARP1, Piao and colleagues (129) showed that silencing PARP1 binding protein (PARBP), a protein that enhances PARP1 activity, sensitized KLM-1 PDA cells to adriamycin, H_2_O_2_, and UV irradiation. Moreover, several studies have demonstrated the radiosensitizing effect of PARP inhibition in PDA cells (130–132). PARP inhibitors are currently in clinical trial for PDA (ClinicalTrials.gov identifiers: NCT00515866, NCT01286987, NCT00047307, NCT01989546, NCT02042378) (123, 133).

**Figure 5:**
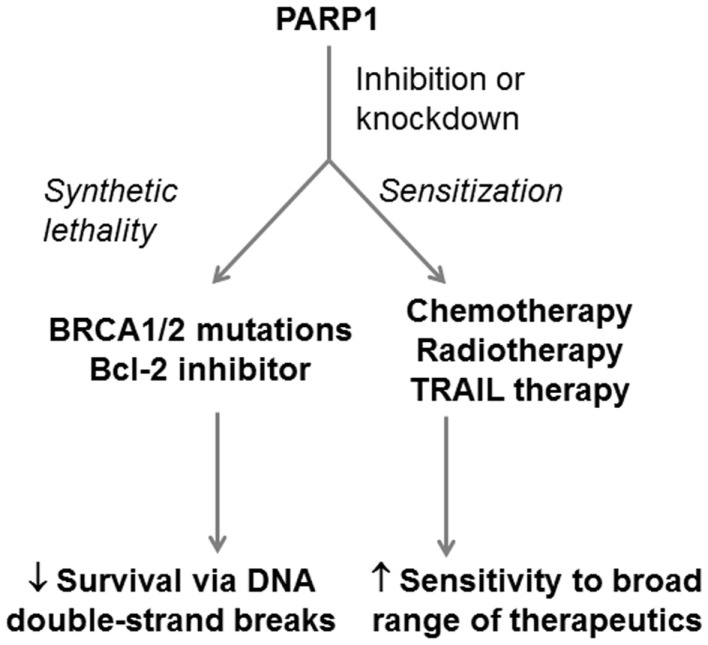
**Potential application of PARP1 as a therapeutic target in PDA**. PARP1 can be targeted in PDA in both synthetic-lethal approaches and in approaches that sensitizPARP1e PDA cells to therapeutics. PARP1 inhibition can be combined with BRCA1/2 defects or Bcl-2 inhibition to induce PDA cell death through double-strand DNA breaks. PARP1 inhibition can also be combined with DNA-damaging therapeutics, radiotherapies, and antibody-based TRAIL therapy to enhance their efficacy.

Poly ADP ribose polymerase 1 is a complex protein with roles in other DNA repair pathways, chromatin remodeling, transcriptional regulation, and cell death pathways (119). This extensive network of interactions means that PARP1 inhibition can affect more than just DNA repair. For example, Yuan et al. (134) found that PARP1 was elevated in pancreatic cancer cell lines resistant to TNF-related apoptosis-inducing ligand (TRAIL) antibody therapy (Panc1 and SUIT2) but low in TRAIL-sensitive lines (MiaPaCa2 and BxPC3). Silencing PARP1 in these resistant lines increased their sensitivity to TRAIL therapy *in vitro* and in a sub-cutaneous animal model (134). Klauschen et al. (135) investigated PARP expression and localization in 178 human PDA tissue specimens, using immunohistochemistry. The patients had undergone surgery for pancreatic masses, without use of chemotherapeutics prior to surgery. The group found that low-level nuclear expression of PARP significantly correlated with reduced median survival (135). As with XRCC1, these results are not necessarily conflicting with the proven effective application of PARP inhibitors in the clinic. A possible explanation is that PARP inhibitors, which inhibit specific functions or regions of PARP, produce very different effects to downregulation of total PARP protein and thus all PARP functions. It again highlights the importance of understanding all of the roles of a BER protein and its overlap with other repair pathways when designing a therapeutic approach. PARP inhibition has proven to be a successful therapeutic approach in many other cancers and is likely to become an effective treatment in synthetic-lethal approaches for PDA (Figure 5).

## Future Directions: The Untapped Potential of Base Excision Repair

The information that we already have on the BER proteins APE1, XRCC1, and PARP1 can be applied to better tailor therapeutic approaches for PDA (Table 2). However, there is a lot of untapped potential for PDA therapeutic targets in this pathway. Glycosylases carry out excision of damaged DNA bases and are possible direct therapeutic targets for the chemosensitization of PDA cells. There are several examples of this in other cancers. For example, MPG inhibition is capable of sensitizing cancer cells to alkylating agents (136). The uracil excision glycosylase SMUG1 carries out the majority of the repair of 5-FU and could be targeted to increase sensitivity to this drug (137). There is also promise in BER proteins that are more downstream in the pathway, for example, inhibition of DNA polymerase beta POLB inhibition has been shown to sensitize cancer cells to the oxaliplatin, cisplatin, and the DNA methylating compound temozolomide (138–140).

**Table 2:** **Summary of APE1, XRCC1, and PARP1 studies in pancreatic cancer**.

Protein	Findings	Reference
	APE knockdown decreased PDA cell proliferation/clonogenicity by inducing apoptosis	(93)
	APE1 inhibition reduced PDA cell proliferation and migration; reduced PDA xenograft growth	(94)
	APE1 inhibition increases Nrf2 activation (potential resistance mechanism to APE1 inhibition)	(96)
APE1	Dual inhibition of APE1 and STAT3 synergistically decreased PDA cell survival and migration	(95)
	APE1 inhibition sensitized PDA cells to gemcitabine	(84)
	APE1 inhibition sensitized PDA cells to gemcitabine	(85)
	APE1 knockdown radiosensitized PDA cells	(86)
XRCC1	XRCC1 Arg399 polymorphism increased PDA risk in smokers XRCC1 Arg399 polymorphism increased PDA risk	(112) (114)
	XRCC1 Arg399 polymorphism reduced PDA patient response to platinum-based therapies	(113)
	BRCA2-defective PDA cells were sensitive to PARP1 inhibitor AG014699 *in vitro* and in PDA xenografts	(124)
	Combined PARP1 and Bcl-2 inhibition synergistically reduced PDA cell tumorgenicity	(125)
	Knockdown of PARP1 binding protein sensitized PDA cells to adriamycin, H_2_O_2_, and UV irradiation	(129)
PARP1	PARP1 inhibition radiosensitized PDA cells	(130–132)
	PARP1 knockdown sensitized PDA cells to TRAIL therapy	(134)
	Low nuclear PARP1 correlated with reduced median survival of PDA patients	(135)

*Studies implicating BER proteins as risk or prognostic factors are highlighted in gray*.

Moving forward, the examples in this review also provide valuable lessons for the effective targeting of BER proteins in therapeutic approaches. BER proteins can have complex roles outside of their primary role in the BER pathway. It is critical to understand these additional functions when deciding how to target a BER protein. Small molecule inhibitors may produce very different effects to knocking-down total protein, by leaving supplementary functions intact. BER proteins may also represent dual targets because of these additional roles, capable of both chemosensitizing cancer cells and interfering with basic survival functions in response to microenvironmental stress; it is essential to understand both the type of DNA damage induced by a chemotherapeutic (there may be multiple forms induced by one chemotherapeutic, either directly or indirectly), as well as the cross-talk between BER and other DNA repair pathways that can respond to the damage. The involvement of XRCC1 in response to platinum-based therapies demonstrates how DNA repair pathways can be linked and can even compete with each other in response to DNA damage (107, 108). By extension, an effective synthetic-lethal approach requires an understanding of how defects in one DNA repair pathway can make BER the Achille’s heal of a cancer cell, as demonstrated by PARP inhibitors. On this point, recent data from the Australian Pancreatic Genome Initiative showed that 14% of pancreatic cancers display high genomic instability and a further 36% display substantial “scattered” genomic instability (141). These have been linked to defects in genes involved in DNA double-strand break repair including BRCA1 and BRCA2 (known to be responsive to PARP1 inhibitors as discussed above) (141). The identification of pancreatic cancer sub-types based on their genomic instability, represents an exciting advance in our knowledge of PDA and could be exploited to personalize synthetic-lethal therapies using BER-targeted approaches.

The paradox in targeting BER and, in fact, any DNA repair pathway is that defects in DNA repair is *responsible* for cancer initiation and progression. How can we be sure that targeting BER proteins to treat one cancer would not generate more aggressive and chemoresistant cancer cells? For a DNA-damaging therapeutic approach to be effective, it must induce enough damage to overcome any advantageous mutations that might arise as a consequence of the treatment – an advantageous mutation is useless if a cancer cell has accumulated so much genomic damage that it cannot maintain the basic functions it requires to stay alive. This is the principle behind DNA-damaging radiotherapies and chemotherapeutics. This is not to say that the recurrence of chemoresistant cancers is not a possibility and it has been documented in multiple cancers in response to DNA-damaging agents (142). Targeting BER proteins in combination with chemotherapeutics can improve the efficacy of existing treatments and potentially reduce the chances of leaving resistant cancer cells behind. Future therapeutic approaches for PDA may involve inhibiting two or more BER proteins responsible for repairing very different types of DNA damage, in combination with multiple structurally diverse chemotherapeutics to maximize genomic damage in PDA cells. If we are to reach that stage, a better understanding of BER proteins in PDA cells and their interactions with other DNA repair and signaling pathways is critical. An equally important consideration is the potential for off-target effects when systemically targeting DNA repair proteins. There is the potential that inhibition of DNA repair proteins could either cause toxicity in normal cells in the body or worse yet, generate an initiating mutation for a cancer elsewhere in the body. This is why a cancer cell-specific approach is preferable when targeting BER proteins.

In summary, the BER pathway holds a lot of potential as a therapeutic target in PDA that is still largely untapped. As we move toward more personalized treatments for patients, BER proteins could be inhibited in combination with existing mutations in cancer cells in synthetic-lethal approaches, in combination with microenvironment-induced stress to reduce cancer cell survival, or in combination with DNA-damaging agents to improve their efficacy. Further studies into the BER pathway in PDA are critical in our search for more effective therapeutic approaches for this devastating disease.

## Author Contributions

GS and PP were involved in the conception, writing, and final approval of this review. JM and DG were involved in interpretation, revision, and final approval of the work. All authors agree to be accountable for all aspects of the review and ensure that questions related to accuracy or integrity of any part of this review are appropriately investigated and resolved.

## Conflict of Interest Statement

The authors declare that the research was conducted in the absence of any commercial or financial relationships that could be construed as a potential conflict of interest.

## Abbreviations

APE1, apurinic/apyrimidinic endonuclease 1; BER, base excision repair; FEN1, flap-endonuclease 1; LIGI, DNA ligase I; LIGIII, DNA ligase III; MBD4, methyl-CpG-binding domain protein 4; MPG, methyl-purine glycosylase; MYH, MutY homologue; NEIL1–3, endonuclease VIII-like 1–3; NTH1, Nth endonuclease III-like 1; OGG1, 8-oxoguanine DNA glycosylase; PanIN, pancreatic intraepithelial neoplasia; PARP1, poly ADP ribose polymerase-1; PDA, pancreatic ductal adenocarcinoma; POLB, DNA polymerase β; SMUG1, single-strand selective monofunctional uracil DNA glycosylase; TDG, thymine DNA glycosylase; TRAIL, TNF-related apoptosis-inducing ligand; UNG, uracil DNA glycosylase; XRCC1, X-ray repair cross-complementing protein 1.
